# When will bioprinting become a therapy? Translational and regulatory challenges in urogenital tissue engineering and surgery

**DOI:** 10.3389/fbioe.2026.1865005

**Published:** 2026-08-13

**Authors:** Margrethe Ruding, Amitha Banu Shajahan, Magdalena Fossum, Cristian Pablo Pennisi, Johan Ulrik Lind

**Affiliations:** 1 Department of Health Technology, Technical University of Denmark, Lyngby, Denmark; 2 Laboratory of Tissue Engineering, Rigshospitalet, Faculty of Health and Medical Sciences, University of Copenhagen, Copenhagen, Denmark; 3 Department of Health Science and Technology, Regenerative Medicine, Faculty of Medicine, Aalborg University, Aalborg, Denmark; 4 Division of Pediatric Surgery, Department of Surgery and Transplantation, Copenhagen University Hospital Rigshospitalet, Copenhagen, Denmark; 5 Laboratory of Tissue Engineering, Department of Women’s and Children’s Health, Karolinska Institutet, Stockholm, Sweden

**Keywords:** autologous, bioprinting, EMA, fda, intraoperative, regulation, tissue engineering

## Introduction

**FIGURE 1:**
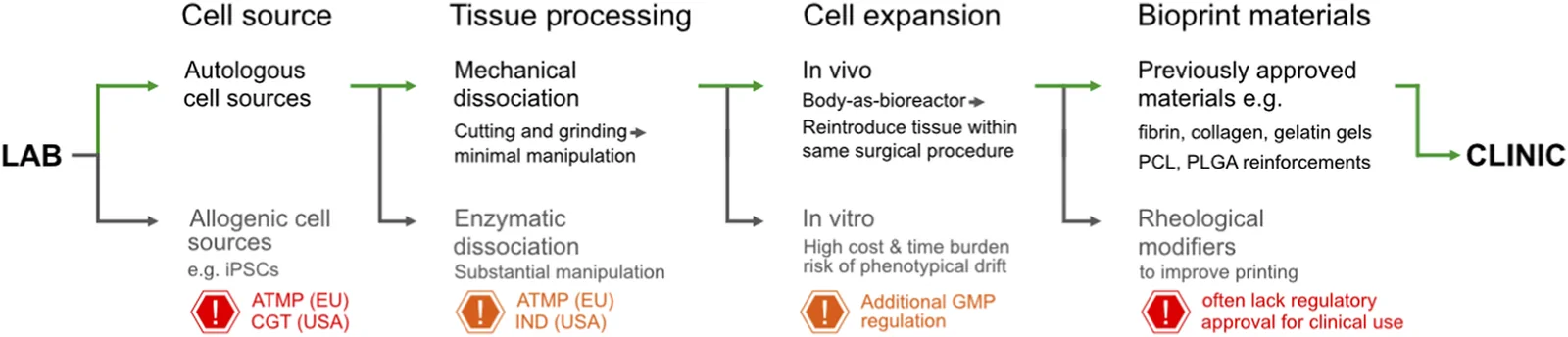
Regulatory roadblocks and opportunities for translating bioprinting into urogenital therapies.

Despite its undeniable promise, bioprinting of human tissues has yet to make an impact in the clinic. We believe that increased attention to regulatory and operational obstacles will be essential for advancing bioprinted therapies beyond early-stage trials (ACTRN12625000088448, 2026; CTRI/2024/11/077162, 2026; CTRI/2024/12/078592, 2026; NCT04925323, 2026; NCT06051747, 2026). This is particularly true within urethral and urogenital tissue engineering and surgery where bioprinted constructs may fall into a “grey zone” between medical devices and advanced biologics. Thus, while several bioprinting strategies have shown promise for bioprinting urinary and genital tissues in the lab (Kim et al., 2019; Yang et al., 2022), as well as in animal models (Barasa et al., 2025; Zhang et al., 2025), none have reached clinical translation (Booth et al., 2024). In our view, bridging this gap will require a shift towards workflows that comply with existing regulatory frameworks. Focusing on European Union (EU) and United States (US) governance, we here offer our opinion on rational choices within i) cell and tissue sources, ii) cell and tissue processing, iii) biomaterials, and iv) practical implementation in surgery, which will serve to make 3D bioprinting directly applicable in urogenital surgeries, see Figure 1.

## Governance overview

To date, no regulatory agency has produced specific guidance for 3D bioprinted therapies. Some countries, including the US and South Korea, have established guidelines for acellular patient-specific medical devices fabricated via additive manufacturing (Ministry of Food and Drug Safety, 2015; U.S. Food and Drug Administration FDA, 2017). Other jurisdictions, such as Japan, have produced broad frameworks for regenerative medicine products that may be applicable to future bioprinted tissues (Pharmaceuticals and Medical Devices Agency, 2014). Consequently, bioprinted interventions are presently regulated under existing tissue-engineering frameworks. Notably, in the EU and US, the regulatory burden is strongly influenced by the degree of manipulation of the underlying biological material.

In the EU, human tissues and cells that are not substantially manipulated, and are used for homologous purposes, are regulated under the Tissues and Cells Directive (2004/23/EC) (European Parliament; Council of the European Union, 2004). The Directive focuses on preventing disease transmission and ensuring traceability and is implemented and overseen by competent authorities in the Member States. By contrast, tissues and cells that are substantially manipulated or intended for non-homologous use are regulated as Advanced Therapy Medicinal Products (ATMPs) (Regulation (EC) No 1394/2007) (European Parliament; Council of the European Union, 2007). ATMPs have very strict good manufacturing practice (GMP) requirements that include donor-to-patient traceability, enhanced control of starting materials and manufacturing environments, and, for ATMPs containing genetically modified cells, long-term patient follow-up of up to 15 years. Consequently, ATMP classification has a major impact on the feasibility, cost, and timeline of clinical translation. In limited cases, certain ATMPs prepared non-routinely for individual patients, under a physician’s responsibility, may be supplied under the Hospital Exemption, subject to authorization and oversight by national competent authorities, and without centralized EMA authorization.

In the US, bioprinted therapies are regulated by the Center for Biologics Evaluation and Research (CBER), within the FDA. CBER regulates biological products, including human cells, tissues, and cellular- or tissue-based products (HCT/Ps). Biological products that are more than minimally manipulated or intended for non-homologous use require authorization through an Investigational New Drug (IND) application prior to clinical trials and compliance with FDA current good manufacturing practice (cGMP) requirements. Like ATMP regulations in the EU, these requirements address traceability, rigorous control of starting materials and manufacturing environments, and, for products containing genetically modified cells, risk-based long-term patient follow-up. However, procedures that involve autologous cells or tissues that are removed from and re-implanted into the same patient during a single surgical procedure, without extensive processing, may fall under the FDA’s Same Surgical Procedure (SSP) exemption (FDA 21 CFR 1271.15(b)) (U.S. Food and Drug Administration, 2019), making them exempt from the most extensive regulations.

## Cell and tissue sources

The cell source is essential in any tissue-engineered and bioprinted therapy. In recent years, patient- or donor-derived induced pluripotent stem cells (iPSCs), terminally differentiated toward specific urogenital cell types, have gained substantial attention as a versatile, seemingly universal option. However, due to concerns over teratoma formation, residual undifferentiated cells, and genetic abnormalities, the use of iPSCs dramatically increases the regulatory burden. In the EU, this will squarely place the product within the tightly monitored ATMP category. In the US, the situation is comparable: the use of iPSCs would classify the construct as a Cellular and Gene Therapy (CGT) product. Thus, while iPSC-derived tissues and organs represent a compelling long-term vision, we believe primary cell sources offer a more direct path to clinically applicable bioprinted therapies.

The most relevant primary urogenital cell types for reconstruction include urothelial cells, stromal fibroblasts, and smooth muscle cells (Xuan et al., 2022). Moreover, epidermal keratinocytes and endothelial cells may also be considered, contributing to epithelial barrier function and vascularization, respectively (Guo et al., 2016; Imbeault et al., 2013). Urothelial cells derived from mucosal biopsies or bladder washings provide the luminal barrier essential for tubular and reservoir structures, while smooth muscle cells harvested from detrusor, ureteral, or corporal tissue supply the contractile and structural functions required for physiological performance (Fossum et al., 2003). Stromal fibroblasts further support extracellular matrix remodeling and tissue stability. While autologous primary cells are not without challenges, including yield, patient variability, and harvesting morbidity, they can be obtained through established surgical procedures, positioning them well within regulatory expectations for low-risk autologous interventions (Juul et al., 2025). However, limited cell yield could pose a critical challenge in the fabrication of large, high-density grafts, which often require *ex vivo* expansion while maintaining cellular integrity.

In addition to cells derived directly from urogenital or other epithelial tissues, a range of complementary autologous cell preparations may be considered, given their substantial regenerative capacity. Stromal vascular fraction, platelet-rich plasma, bone-marrow aspirate concentrate, and micro-fragmented adipose tissue can supply angiogenic, immunomodulatory, and matrix-remodeling signals and may be further prepared using minimally manipulative techniques such as centrifugation, washing, and micro-fragmentation (Perini et al., 2025). As these cell preparations are derived from adipose tissue or peripheral blood that is accessible during surgical procedures, the operative complexity of obtaining the cells remains low (Rizkalla et al., 2025). These complementary cell sources are therefore attractive for a range of regenerative medicine applications, including bioprinted urogenital interventions.

## Cell and tissue processing

Cell processing and preparation may be as critical as cell sourcing to develop clinically applicable bioprinting. To be compatible with extrusion- or inkjet-based bioprinting, primary tissue must be processed into single-cell suspensions or tissue fragments. Since bioprinting is constrained to sub-physiological cell concentrations, cell yield and proliferative capacity can become critical limitations. To address these constraints, *in vitro* tissue culture and cell expansion are often employed in tissue engineering and bioprinting. However, such culture steps can significantly impede clinical adoption. In practice, *in vitro* culture may require several weeks to months, and substantial manual labor (Griffith and Naughton, 2002; Hunsberger et al., 2015). Beyond the associated costs and time burdens, *in vitro* culture introduces regulatory challenges to ensure GMP compliance and avoid phenotypic drift (Pettersson et al., 2024). For these reasons, we believe that—when possible—approaches based on autologous cell sources without *ex vivo* culture steps offer the most immediate route to clinical application.

Beyond cell culture, the full tissue-processing workflow must be considered. For instance, bioprinting workflows may be designed to fall under the same surgical procedure (SSP) frameworks in the US and EU. In the US, SSP eligibility requires autologous tissue to be re-implanted into the same patient during a single procedure and subjected only to minimal manipulation. In the EU, similar principles apply under Regulation (EC) No 1394/2007 (European Parliament; Council of the European Union, 2007), emphasizing autologous use within the same procedure and the absence of substantial manipulation. Although eligibility is assessed case-by-case by national authorities, enzymatic or chemical digestion of primary tissues, commonly used to generate printable cell suspensions, must likely be avoided in SPP-compatible procedures.

While SSP exemptions have not yet been applied to bioprinted urogenital therapies, encouraging examples exist in more conventional tissue-engineering. One example is perioperative layered autologous tissue expansion (PLATE) grafts (Willacy et al., 2024). PLATE grafts consist of mechanically dissociated primary urethral tissue embedded within compressed collagen sheets. These grafts are created directly in the operating room and do not require *in vitro* culture steps. Unlike traditional approaches that rely on *ex vivo* cell culture (Barasa et al., 2025; Chapple, 2019), this method offers a single-stage alternative that has been validated in animal models (Chamorro et al., 2022; Juul et al., 2023; Willacy et al., 2024). By relying on autologous cell sources, avoiding extensive cell manipulation, and using well-characterized biomaterials, PLATE grafts for vaginal atresia have been allowed to progress under national regulatory interpretations (ISRCTN12529893, 2026). Bioprinting approaches that adopt a similar logic—minimally manipulated autologous cell sources, established biomaterials, and intraoperative implementation—may therefore, in principle, fall within SSP frameworks. A central challenge in adapting such procedures for bioprinting is the generation of printable autologous cell suspensions without enzymatic tissue digestion. One promising route may be the direct incorporation of micro-fragmented tissues as a cell source within bioinks.

## Biomaterials

Biomaterial selection plays a multifaceted role in 3D bioprinting for clinical use. It is crucial for achieving appropriate rheological and mechanical properties before, during, and after extrusion, for supporting tissue development, as well as for host tissue response. Additionally, regulatory approval must be considered.

At present, it is unclear how the addition of bioink materials or even the printing process itself will influence regulatory classification. Regardless, biomaterials with prior regulatory approval and established use in soft, compliant, and implantable devices are the most obvious choices. For instance, natural polymers such as collagen, fibrin, and gelatin are widely utilized in FDA-cleared wound matrices and absorbable hemostats and provide biologically favorable substrates that support epithelial and smooth muscle cell integration. Hyaluronic acid, approved for injectable and urological applications, likewise offers adjustable viscosity, biocompatibility, and relevance to mucosal tissues (Benwood et al., 2021).

Beyond the biocompatibility of the non-cellular bioink components, printability, post-extrusion gelation and crosslinking must be carefully considered. Within the bioprinting community, these challenges are addressed through a range of strategies. Rheological modifiers, including thixotropic agents, are used to increase bioink viscosity and enhance shear-thinning behavior; UV- or enzyme-mediated crosslinking is frequently applied to promote longer-term hydrogel stability. However, auxiliary components without regulatory approval for clinical use hinder clinical translation, as safety must first be established.

Finally, while soft, degradable hydrogels support cell viability, proliferation, and tissue regeneration, they lack the mechanical robustness required for surgical handling, suturing, and implantation into highly mobile urogenital tissues. This may necessitate the incorporation of mechanically reinforced elements. These may be introduced via resorbable synthetic polymers, such as polycaprolactone and polylactic acid, in multimaterial printing strategies. Despite established safety profiles, their use in bioprinted urogenital constructs requires validation of mechanical durability, degradation behavior, and sterility to meet regulatory expectations.

## Practical implementation

Bioprinted autologous tissue products, created during surgeries, are challenged by patient-specific variability and operator-dependent processing. Reproducibility, quality assurance, sterility validation, and GMP implementation are thus major barriers. Because intraoperative workflows offer limited options for real-time characterization, key steps must be standardized and pre-validated. A critical step may thus be the development of rapid, instrument- or computer-assisted quality controls, that can be implemented seamlessly as part of the procedure. Further, because printing occurs within or adjacent to an open surgical field, hardware and workflows must be designed to ensure sterility, e.g., through closed-system mixing, sterile-draped printing modules, and aseptic tool-to-tissue interfaces. Bioprinting hardware and automation innovations will thus play a central role, but while automation may reduce operator-dependent variability, integration of bioprinting within surgeries will also require tight coordination and training of surgeons, engineers, and nursing staff.

## Summary

At present, neither the FDA nor the EMA has issued dedicated guidelines on the clinical use of bioprinting in regenerative medicine. Until regulatory frameworks evolve to accommodate bioprinted therapies, it remains unclear how the printing procedures will influence regulatory classification. Meanwhile, we believe the most immediate route toward clinical implementation in urogenital surgery is to design workflows based on minimally manipulated autologous tissues and cell sources. This calls for increased focus on the development of intraoperative bioprinting procedures compatible with SSP frameworks, as well as automated quality control. Ultimately, meaningful progress will depend on coordinated efforts among regulators, clinicians, scientists, and engineers to translate technological advances into safe, clinically as well as economically viable solutions that meet the needs of the patient communities we aim to serve.
